# Expression and Evolution of Short Wavelength Sensitive Opsins in Colugos: A Nocturnal Lineage That Informs Debate on Primate Origins

**DOI:** 10.1007/s11692-013-9230-y

**Published:** 2013-04-17

**Authors:** Gillian L. Moritz, Norman T.-L. Lim, Maureen Neitz, Leo Peichl, Nathaniel J. Dominy

**Affiliations:** 1 Department of Biological Sciences, Dartmouth College, The Class of 1978 Life Sciences Center, 78 College Street, Hanover, NH 03755 USA; 2 Department of Wildlife, Fish and Conservation Biology, University of California, One Shields Avenue, Davis, CA 95616 USA; 3 Department of Ophthalmology, University of Washington, Box 356485, 1959 NE Pacific Street, Seattle, WA 98195 USA; 4 Max Planck Institute for Brain Research, Deutschordenstrasse 46, 60528 Frankfurt am Main, Germany; 5 Department of Anthropology, Dartmouth College, 6047 Silsby Hall, Hanover, NH 03755 USA

**Keywords:** Retina, Visual pigment, Dermoptera, Scandentia, Primates

## Abstract

A nocturnal activity pattern is central to almost all hypotheses on the adaptive origins of primates. This enduring view has been challenged in recent years on the basis of variation in the opsin genes of nocturnal primates. A correspondence between the opsin genes and activity patterns of species in Euarchonta—the superordinal group that includes the orders Primates, Dermoptera (colugos), and Scandentia (treeshrews)—could prove instructive, yet the basic biology of the dermopteran visual system is practically unknown. Here we show that the eye of the Sunda colugo (*Galeopterus variegatus*) lacks a tapetum lucidum and has an avascular retina, and we report on the expression and spectral sensitivity of cone photopigments. We found that Sunda colugos have intact short wavelength sensitive (S-) and long wavelength sensitive (L-) opsin genes, and that both opsins are expressed in cone photoreceptors of the retina. The inferred peak spectral sensitivities are 451 and 562 nm, respectively. In line with adaptation to nocturnal vision, cone densities are low. Surprisingly, a majority of S-cones coexpress some L-opsin. We also show that the ratio of rates of nonsynonymous to synonymous substitutions of exon 1 of the S-opsin gene is indicative of purifying selection. Taken together, our results suggest that natural selection has favored a functional S-opsin in a nocturnal lineage for at least 45 million years. Accordingly, a nocturnal activity pattern remains the most likely ancestral character state of euprimates.

**Electronic supplementary material:**

The online version of this article (doi:10.1007/s11692-013-9230-y) contains supplementary material, which is available to authorized users.

## Introduction

All organisms must obtain information about their physical environment and respond to changing conditions such as circadian variation in light intensity and spectral composition. For most vertebrates, image-forming vision is central to survival, underlying fundamental behaviors such as predator avoidance, foraging, and mate recognition. Thus, it stands to reason that the genes that encode vision-mediating proteins are under strong selective pressure, and that molecular changes to these genes are ultimately advantageous to the survival of the organism (Davies 2011). In fact, molecular adaptations to diverse light environments are prevalent across vertebrates, determining the number, type, and spectral sensitivities of visual pigments. This strong link between a visual system and its predominant photic environment has practical use for inferring ancestral behaviors, including the origin and evolution of each major vertebrate radiation (Davies et al. 2012).

The dichromatic color vision of most mammals is based on the presence of two cone opsins—the long wavelength sensitive (L-) opsin and the short wavelength sensitive (S-) opsin (Peichl 2005). A puzzling exception to this pattern is the loss of the S-opsin, and hence the absence of color vision, or monochromatic vision, in a number of—mostly nocturnal—mammals (review: Jacobs 2013). It has been tempting to link the L-cone monochromacy of these species with dark (scotopic) light environments, and to assume that relaxation of natural selection is the cause of disabling mutations on the S-opsin gene. This hypothesis is weakened in part by variation among closely related species with similar scotopic behaviors; for instance, the Syrian golden hamster, *Mesocricetus auratus*, is an L-cone monochromat, whereas the Siberian dwarf hamster, *Phodopus sungorus*, has both opsins intact (Calderone and Jacobs 1999). Among bats, the S-opsin gene has been lost in some lineages, while both cone opsin genes have been maintained over many millions of years in others (Wang et al. 2004; Müller and Peichl 2005; Müller et al. 2007, 2009; Zhao et al. 2009a, b). This latter finding suggests that dichromatic vision is advantageous for some nocturnal species; however, the functional and adaptive significance of such vision is uncertain. Importantly, a similar pattern has been reported among nocturnal primates and it has had the effect of fueling debate focused on primate origins.

### Euprimate Origins

The common ancestor of euprimates (primates of modern aspect or crown-clade primates) is often reconstructed as nocturnal (Martin 1990; Heesy and Ross 2001; Ravosa and Savakova 2004; Martin and Ross 2005; Ravosa and Dagosto 2007; Ross and Kirk 2007). As a result, a nocturnal activity pattern is central to almost all hypotheses on the adaptive origins of primates (Cartmill 1992). This enduring view has been challenged in recent years on the basis of variation in the cone opsin genes of nocturnal primates (Tan et al. 2005). Specifically, some taxa—the lorisoids, dwarf and fork-marked lemurs, and night monkeys—have lost the functionality of their S-opsin genes and possess monochromatic cone vision (Wikler and Rakic 1990; Jacobs et al. 1996a; Tan et al. 2005; Veilleux et al. 2013). In other lineages—the mouse lemurs, *Avahi*, *Lepilemur*, aye–ayes, and tarsiers—the S- and L-opsin genes are intact (Tan and Li 1999; Tan et al. 2005; Melin et al. 2013). The expression of both visual pigments has been verified in two of these taxa (Hendrickson et al. 2000; Dkhissi-Benyahya et al. 2001; Peichl et al. 2001), indicating the potential for dichromatic color vision.

On the assumption that monochromatic vision is favored under nocturnal conditions, Tan et al. (2005) suggested that the common ancestor of euprimates was either cathemeral (arrhythmic) or diurnal, and that living primates represent at least seven independent shifts to nocturnality. Furthermore, some of these shifts must have been relatively recent because genetic drift has yet to result in disabling mutations of the S-opsin genes (Tan et al. 2005). The hypothesis that intact S-opsin genes are functionless anachronisms for all nocturnal species is challenged by evidence of purifying selection at the S-opsin gene locus of tarsiers (Kawamura and Kubotera 2004) and aye–ayes (Perry et al. 2007). These findings indicate that at least some nocturnal primate species benefit from having an intact S-opsin gene, although the functional advantages are uncertain (Melin et al. 2012). Jacobs (2008) has given a critical overview of this controversy.

If the behavior of tarsiers (*Tarsius*) can be inferred from orbital morphology, the hyper-enlarged orbits of *Tarsius eocaenus* from the Middle Eocene suggests that the S-opsin genes of tarsiers have survived 45 million years of nocturnality intact (Rossie et al. 2006). Such antiquity of color vision genes at the genus taxonomic level is seldom paralleled. Thus inferences on the evolution and function of S-opsin genes, and therefore the origins of primates, can be tenuous. Yet the fossil record of the superordinal group Euarchonta—the orders Primates, Dermoptera (colugos), and Scandentia (treeshrews)—is instructive, and it suggests that extant colugos are ‘living fossils’ that have scarcely changed since the Middle Eocene (Ducrocq et al. 1992; Marivaux et al. 2006). The implication of longstanding nocturnality in this lineage agrees well with the nuclear architecture of their rods (Solovei et al. 2009; Perry and Pickrell 2010), and motivates the present study of their opsins.

### Colugos: Systematics, Anatomy, and Behavior

**Fig. 1:**
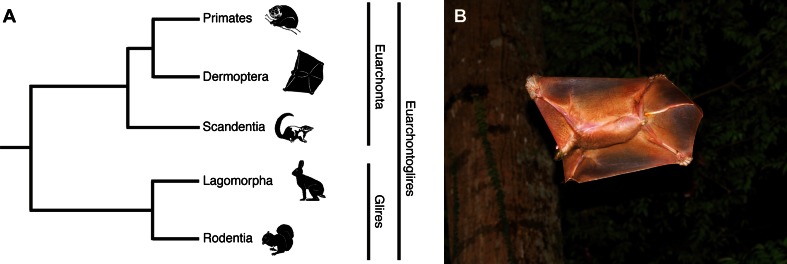
**a** Phyletic position of colugos (Dermoptera) within the grandorder Euarchonta and superorder Euarchontoglires. Modified from Martin (2008). **b** Sunda colugo (*Galeopterus variegatus*) in the Bukit Timah Nature Reserve, Singapore (photograph by Norman T.-L. Lim)

Although the internal structure of Euarchonta is unresolved (Martin 2008), most recent evidence favors a sister grouping between colugos and primates (Fig. 1a; Janečka et al. 2007; Meredith et al. 2011; Perelman et al. 2011) or the concept of Sundatheria (Dermoptera + Scandentia) as the sister taxon to Primates (Bloch et al. 2007; O’Leary et al. 2013). Presently, there are two recognized species of colugo, now in separate genera: the Sunda colugo *Galeopterus variegatus* (formerly *Cynocephalus variegatus*) and the Philippine colugo *Cynocephalus volans* (Janečka et al. 2008). Both species are nocturnal. A distinctive feature of colugos is the large gliding membrane (patagium) that practically surrounds the entire body. As a result, colugos are sometimes called ‘flying lemurs’, but—as Simpson (1945) noted wryly—they “are not lemurs and [they] cannot fly”. Colugos differ from other gliding mammals in that the patagium extends between the hind limbs and the short tail (Fig. 1b), including even the fingers and toes (Beard 1993).

They also possess serrated upper incisors and procumbent lower incisors that are uniquely subdivided into as many as 15 comb-like tines (Rose et al. 1981). The function of these tines is uncertain, but they appear to support grooming and foraging behaviors (Aimi and Inagaki 1988). The diet of colugos consists mainly of leaves, to which they have corresponding digestive adaptations (Wischusen et al. 1994). In general, the behavioral ecology of colugos is poorly known, although recent field studies are yielding valuable new information (Wischusen and Richmond 1998; Mendoza and Custodio 2000; Agoramoorthy et al. 2006; Lim 2007; Dzulhelmi and Abdullah 2009; Byrnes et al. 2008, 2011a, b).

Colugos are thus unique nocturnal mammals that provide an important comparative context for testing hypotheses related to the color vision and origins of primates. Here we report on the expression and inferred spectral sensitivity of cone photopigments in the retinae of Sunda colugos (*G. variegatus*). Our results fill a crucial phylogenetic void and provide new insight into how and why S-opsin genes are maintained in a nocturnal milieu.

## Materials and Methods

### Animals and Tissue Preparation

A population of Sunda colugos inhabits the forests of Singapore (Lim and Ng 2010; Lim et al. 2013) and animals are occasionally found dead by members of the public and donated to the Raffles Museum of Biodiversity Research, National University of Singapore. Here we report on the retinal histology and opsins of three such animals, two adult males and a juvenile female (accession nos. ZRC.4.8122, ZRC.4.8138, ZRC.4.8186, respectively). Tissue preservation varied as a function of how soon the eyes were enucleated and fixed post mortem. Our best results were obtained from eyes that were fixed within hours of death (e.g., ZRC.4.8138). In each case, the eyes were marked at their dorsal pole for orientation and cut slightly open at the corneal rim for better fixative penetration, and fixed in conventional 10 % formalin in physiological saline for several days to several weeks. The eyes were then transferred to 0.1 M phosphate buffer (PB, pH 7.4) with 0.05 % sodium azide and stored at 4 °C until processing. Eye dimensions were recorded before the eye was cut fully open around the cornea. The cornea, lens and vitreous were removed, and the retina was carefully isolated from the eyecup.

### Retinal Histology and Opsin Immunohistochemistry

For assessment of general retinal morphology, pieces of retina were embedded in Epon, sectioned vertically (i.e. perpendicular to the retinal layers) at 1 μm, and stained with toluidine blue. For assessment of retinal vascularization, a retinal segment measuring ca. 5 × 5 mm and containing the optic nerve head was stained with a 3,3′-diaminobenzidine (DAB) reaction to selectively visualize the endogenous peroxidase in the vasculature. Following the protocol of Peichl (1992), the retinal piece was washed in PB and then incubated in a solution of 0.05 % DAB and 0.01 % hydrogen peroxide in PB until the peroxidase reaction was fully developed (ca. 10 min). The reaction was stopped by several washes in PB; the tissue was then flat-mounted on a slide and coverslipped with an aqueous mounting medium (AquaPoly/Mount, Polysciences Inc., Warrington, USA).

Immunohistochemistry was performed on frozen sections and on unsectioned retinal pieces. The tissue was cryoprotected by successive immersion in 10, 20 and 30 % (w/v) sucrose in PB. For sections, the retina was transferred from 30 % sucrose to tissue-freezing medium (Reichert-Jung, Bensheim, Germany), sectioned vertically at a thickness of 14 μm with a cryostat, and collected on SuperFrost slides (Menzel, Braunschweig, Germany). For staining of unsectioned pieces, the tissue was repeatedly shock-frozen and thawed to improve penetration of the antibodies.

Rod opsin (rhodopsin) was detected with the mouse monoclonal antibody rho4D2 (dilution 1:500) provided by R. S. Molday (Hicks and Molday 1986). The S-opsin was detected with the rabbit antiserum JH 455 (dilution 1:5,000) or the goat antiserum sc-14363 (dilution 1:100 or 1:500; Santa Cruz Biotechnology, Heidelberg, Germany), whereas the L-opsin was detected with the rabbit antiserum JH 492 (dilution 1:2,000). The rabbit cone opsin antisera were provided by J. Nathans (Wang et al. 1992). Opsin immunolabeling followed protocols described in Peichl et al. (2005) and Schleich et al. (2010). A brief description of the procedure and controls is given in the Supplemental Material.

### Analysis

Tissue was analyzed with a Zeiss Axioplan 2 microscope equipped with epifluorescence. Micrographs were taken with a CCD camera and the Axiovision LE software (Carl Zeiss Vision, Germany). The images were adjusted for brightness and contrast using Adobe Photoshop 7.0. Rod densities were estimated from outer nuclear layer (ONL) soma counts in vertical cryostat sections labeled for the rod opsin. The density of cones expressing S- and L-opsins in various retinal regions was assessed in PAP-DAB-reacted pieces labeled by JH 492 or JH 455. Counts were made in sample fields with a ×40 or ×63 objective focused on the cone somata labeled by these antisera. This process gave reliable counts in regions where some of the outer segments had been lost due to suboptimal preservation of the retina. No attempt was made to obtain detailed maps of cone topographies. Cone and rod densities were not corrected for shrinkage, which was negligible in the tissues mounted with the aqueous medium.

### Cone Opsin Spectral Tuning Sites

To estimate the spectral sensitivities (λ_max_) of the S- and L-opsins, genomic DNA was extracted from the muscle tissue of male ZRC.4.8122 following the methods of Neitz et al. (1991). Among vertebrates, the λ_max_ of the S-opsin is determined by residues present at seven sites (46, 49, 52, 86, 93, 114 and 118) (Shi et al. 2001). In most mammals, a λ_max_ in the ultraviolet (UV, 360–400 nm) or violet-blue (400–450 nm) region of the spectrum is determined by the residue at site 86, with Phe (Phe86) conferring UV sensitivity and either Leu, Ser, Tyr or Val causing a shift to longer wavelengths (~415–440 nm) (Hunt et al. 2004, 2007, 2009). The aye–aye (*Daubentonia madagascariensis*) is an exception to this pattern; an amino acid change from Thr to Pro at site 93 (denoted Thr93Pro) partly overrides the spectral effects of Phe86, resulting in an intermediate λ_max_ of ~406 nm (Carvalho et al. 2012).

Similarly, the λ_max_ of the L-opsin pigment can be predicted by residues present at three sites: site 180 encoded by exon 3 and sites 277 and 285 encoded by exon 5 of the L-opsin gene (Neitz et al. 1991). Pigments with the hydroxyl-bearing amino acids (Ser, Tyr, Thr) at sites 180, 277 and 285 have λ_max_ values at relatively longer wavelengths than the corresponding pigments with non-polar amino acids (Ala, Phe, Ala) (Neitz et al. 1991; Merbs and Nathans 1992; Asenjo et al. 1994; Yokoyama and Radlwimmer 2001). The magnitudes of the shifts conferred by the amino acid at position 180 depends on what amino acids are present at the other positions (Neitz and Neitz 2011).

### Partial Gene Sequences

The S-opsin gene was amplified using degenerate primers described previously by Wang et al. (2004) for amplifying the S-opsin genes of bats. Two segments were amplified, Exons 1–4 and Exons 4–5. The colugo S-opsin exon 1–4 segment was amplified in hot start PCR using the ABI Gene Amp XL PCR kit and AmpliWax Gems (ABI, Foster City, USA). The final reaction volume was 100 μl, with final concentrations of 1.5 mM magnesium acetate, 200 μM for each dNTP, and 900 nM for each primer. The PCR product was gel purified and ligated into the pCR2.1 cloning vector using the TA Cloning Kit (Invitrogen, Carlsbad, USA). Multiple individual clones were sequenced using Big Dye Terminator V3.1, and the sequences were used to design colugo-specific S-opsin primers (colugoSFwd1: 5′GCCTTCATGGGCTTTGTCTTCT; colugoSRev1: 5′CCCCCATCATTCCCTTTCAGTA; colugoSFwd2: 5′TGGGGAAAAGGAGTTTGGTTCT; colugoSRev2: 5′CTGGCTATGCACATTTCCAGGT). These primers were used to amplify segments of the S-opsin gene from genomic DNA and the PCR products were directly sequenced. Amplifications using the primer pair colugoSFwd1 and colugoSRev1, were in a final reaction volume of 50 μl, using the ABI AmpliTaq Gold kit. The final concentrations were 1.0 mM MgCl_2_, 125 μM for each of the dNTPs, and 600 nM for each primer. Reactions were incubated at 95 °C for 9 min followed by 35 cycles of 94 °C for 30 s, 64 °C for 30 s, and 72 °C for 30 s. Finally, the reactions were incubated at 72 °C for 7 min. The same parameters were used for reactions with the primers colugoSFwd2 and colugoSRev2 except that following the initial incubation at 95 °C for 9 min, reactions were subjected to 35 cycles of 94 °C for 30 s, 64 °C for 30 s, and 72 °C for 2 min. PCR products were directly sequenced.

The colugo S-opsin gene exon 4–5 segment was amplified using primers that were described previously (Wang et al. 2004). Each PCR reaction was in a final volume of 50 μl. Final concentrations and cycling parameters were the same as those described above for primers colugoSFwd1 and colugoSRev1. The PCR product was directly sequenced with the same primers used to amplify it.

Exon 5 of the colugo L-opsin gene was amplified using the ABI AmpliTaq Gold kit. The primers were tcOp5 Fwd 5′GAATCCACCCAGAAGGCAGAG and tcOp5 Rev 5′ACGGGGTTGTAGATAGTGGCA. The final reaction volume was 50 μl with a final concentration of 1.0 mM MgCl2, 125 μM for each of the dNTPs, and 600 nm each for the forward and reverse primers. Samples were initially incubated at 95 °C for 9 min followed by 35 cycles of 94° for 15 s, 59° for 15 s and 72 °C for 30 s. The reactions were then incubated at 72 °C for 10 min.

PCR products were prepared for sequencing using Centricon 30 filters (Millipore) according to the manufacturer’s recommendations. The Big Dye Terminator V3.1 kit (ABI) was used for sequencing, and the reaction products were analyzed on an ABI 3100 capillary sequencer.

### Ancestral State Reconstruction

Comparative sequence data for euarchontans was obtained from GenBank and aligned via ClustalW in MEGA5 (Tamura et al. 2011). The evolutionary history of euarchontan S-opsin genes was inferred using the Neighbor-Joining (NJ) method (Saitou and Nei 1987). The NJ tree and others based on Bayesian estimates of phylogeny from the 10kTrees website (Arnold et al. 2010) were used to construct a composite tree topology for the following species: *G. variegatus* KC865781; *Tupaia belangeri* EU487780; *Bos taurus* AH003442; *Daubentonia madagasariensis* EF667285; *Avahi laniger* DQ191893; *Propithecus verreauxi* DQ191935; *Microcebus murinus* DQ191922; *Eulemur fulvus* AB111464; *Tarsius bancanus* AB111463; *Tarsius syrichta* DQ191954; *Cebus olivaceus* AH005810; *Pan troglodytes* NM_001009127; *Homo sapiens* U53874; *Mus musculus* AH005191; *Rattus norvegicus* NM_031015. Ancestral states were inferred using the Maximum Likelihood method (Nei and Kumar 2000) under the JTT matrix-based model (Jones et al. 1992) in MEGA5 (Tamura et al. 2011).

### S-Opsin Gene Sequence Analysis

Evolutionary patterns of selective constraint on the S-opsin gene (exon 1) were assessed using the Selecton server (Stern et al. 2007) and the Codeml package in PAML (v4.4) (Yang 1997). PAML uses a maximum likelihood framework to estimate the ratio of non-synonymous to synonymous rates (d_N_/d_S_ or ω) for the entire data set and tree (M0 model), for particular lineages (branch models), for particular codons (sites models), or for particular codons within particular lineages (branch-site models). The ω value is thought to reflect the degree of evolutionary constraint, or selection pressure on the site class and/or lineage of interest, with ω < 1 indicating purifying selection, ω = 1 neutral evolution, and ω > 1 positive selection. To test for the possibility of branch-specific ω > 1, a model in which all branches in the tree are assigned the same ω (M0) was compared with a model in which specific branches have a different ω value (two-ratio) (Yang and Nielsen 1998). Models incorporating site classes under positive selection (M2a) were also compared to neutral models (M1a). We calculated the probability that two models should differ in log likelihood as much as observed, with the test statistics following a x^2^ distribution and degrees of freedom equal to the difference in the number of parameters between the two models.

## Results

### General Features of the Eye and Retina

**Fig. 2:**
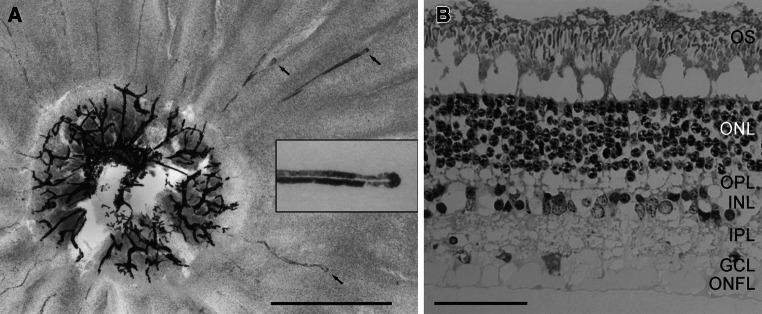
**a** Optic disc region of the colugo retina in flat view. Blood vessels were labeled by a DAB reaction. The papilla is densely vascularized, but only a few capillaries extend outside the papilla and into the retina for 1–2 mm. These capillaries form hairpin loops to return to the papilla (three loops arrowed). The remainder of the retina is avascular. The *inset* shows the hairpin turn of one arrowed capillary at higher magnification. **b** Colugo retinal layering. Transverse semithin section stained with toluidine blue. Tissue preservation is not perfect, as indicated by the large vacuoles between OS and ONL and in other layers.* OS* photoreceptor outer segments,* ONL* outer nuclear layer,* OPL* outer plexiform layer,* INL* inner nuclear layer,* IPL* inner plexiform layer,* GCL* ganglion cell layer,* ONFL* optic nerve fiber layer. *Scale bars* (**a**) 1 mm; (**b**) 50 μm

General features of the eye and retina are reported in Table 1 and as Supplemental Material. Inspection of the fundus in opened eyecups revealed no obvious blood vessels emerging from the optic disc or extending across the retina. Hence we stained a piece of central retina including the optic nerve head with a DAB reaction to label blood vessels. The papilla (optic disc) contained many smaller blood vessels, but only a few capillaries extended outside the papilla and into the retina for 1–2 mm. These capillaries formed hairpin loops to return to the papilla (Fig. 2a). The remainder of the colugo retina was avascular. When the retina was removed, the remaining fundus showed a uniformly dark pigmentation. There was no indication of a typical choroidal tapetum lucidum lining the fundus.

**Table 1:** Eye and photoreceptor properties of the Sunda colugo, *G. variegatus*

General features	
Body weight (kg)	0.9–1.3
Eye axial length (mm); n = 3	15.5/15.7/16.4
Eye equatorial diameter (mm)	16.5/17.4/19.0
Corneal diameter (mm)	13.0/14.0/14.7
Lens diameter (mm)	9.0/10.0/10.8
Lens thickness (mm)	6.4/8.3/8.0
Photoreceptors	
Rod density estimate (mm^−2^)	200,000–250,000
Cone density (mm^−2^)	~2,500–11,800
Cone %age of photoreceptors	1–5
Density of L-opsin-expressing cones (mm^−2^)	~2,400–11,700
Density of S-opsin-expressing cones (mm^−2^)	~100–480
S-cone %age of cones	2–9

Transverse semithin sections of the retina showed the conventional layering of mammalian retinae (Fig. 2b). The ONL contained 6–8 tiers of photoreceptor somata and was the thickest of the layers, which is typical for the rod-dominated retinae of nocturnal mammals. Total retinal thickness was about 170 μm, as measured in cryostat sections of mid-peripheral to peripheral retina. Because of the tissue shrinkage during Epon embedding, the section shown in Fig. 2b appears somewhat thinner.

### Photoreceptor Characteristics

**Fig. 3:**
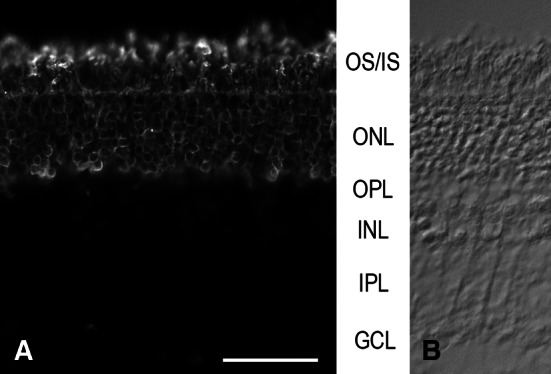
**a** Transverse cryostat section immunolabeled for rod opsin. There is a dense population of rods that fill the entire ONL with their somata. **b** Phase image of part of the field in A to show the retinal layers. OS/IS, photoreceptor outer and inner segments; other abbreviations as in Fig. 2. *Scale bar* 50 μm

The rod dominance is obvious in sections where the rods are labeled by a rod opsin-specific antibody (Fig. 3). As common in mammals, the rod opsin antibody rho4D2 labeled the entire photoreceptors including their somata. The latter fill the ONL, and the rod outer segments form a continuous lawn at the outer retinal surface. Rod densities were estimated from counts of rod somata in vertical cryostat sections of mid-peripheral retina, immunolabeled for rod opsin. They were in the range of 200,000–250,000 mm^−2^. With cone densities in the range of 2,500–11,800 mm^−2^ (see below and Table 1), estimated total photoreceptor densities are about 203,000–262,000 mm^−2^. Accordingly, rods make up 95–99 % of colugo photoreceptors, which is characteristic of nocturnal mammals. However, due to the limited thickness of avascular retinae, the absolute rod density is uncharacteristically low for a nocturnal mammal (see “Discussion” and Supplemental Material).

The colugo retina contains a consistent population of cones, comprising 1–5 % of the photoreceptors. In the temporal quadrant, cone densities were between 2,500 and 5,400 mm^−2^, with no clear peak that would indicate a central area, nor a consistent central-peripheral density gradient. In sample fields from dorsal periphery, cone densities were 7,500–8,000 mm^−2^, and in fields from ventral periphery 7,800–11,800 mm^−2^. Hence it appears that colugo cone densities are higher in peripheral than in central retina, albeit the available tissue did not allow compilation of a full topographical map of cone densities.

**Fig. 4:**
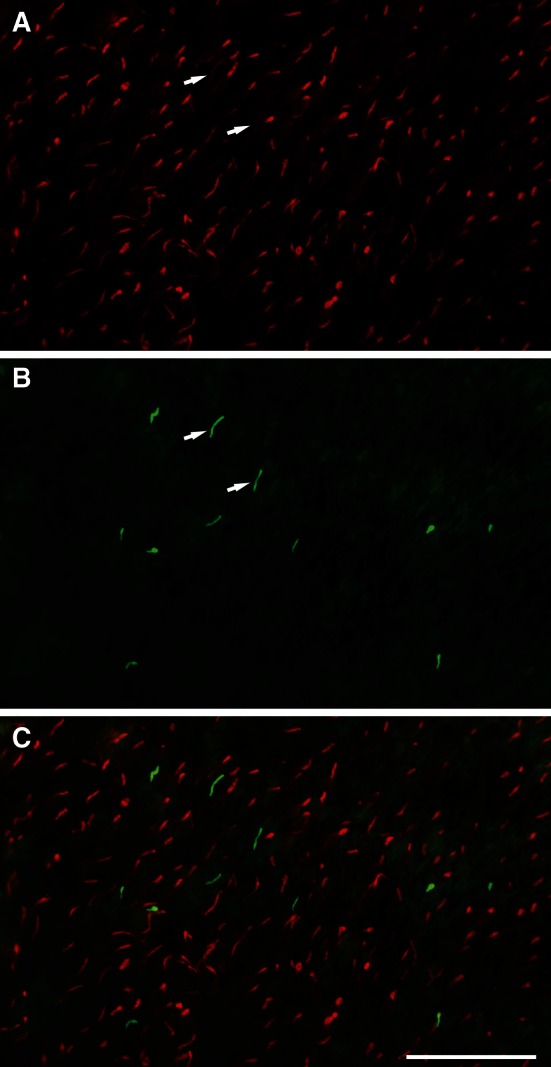
Double immunofluorescence labeling for S- and L-opsin in a flatmounted piece of ventral peripheral retina. **a** L-opsin labeling shows a substantial population of cones, the focus is on the outer segments. **b** S-opsin labeling shows a very sparse population of cones. Each of the S-opsin-expressing outer segments coexpresses some L-opsin (*two examples arrowed* in **a** and **b**). **c** Merge of the images in (**a**) and (**b**). *Scale bar* 50 μm

A large majority of cones expressed the L-opsin (Fig. 4; Supplemental Fig. S1 & S2). Sampling the densities of S-opsin-expressing cones at various retinal locations yielded about 100–480 mm^−2^, depending on region. Higher densities were in the mid-peripheral retina. Comparison of L-cone densities and S-cone densities at neighboring positions (cf. Supplemental Fig. S1) showed S-cone proportions of 4–5 % of the cones near the papilla, of 6–9 % in midperipheral retina, and of 2 % in ventral periphery. Unexpectedly, double immunofluorescence revealed that most cones expressing the S-opsin also coexpressed some L-opsin (Fig. 4). Only a small proportion of S-cones were ‘pure’ or devoid of L-opsin expression (Supplemental Fig. S2). Hence, the colugo retina contains a majority of pure L-cones, a minority of dual-pigment cones, and a sparse population of pure S-cones. The available material did not allow quantification of the proportion and distribution of pure S-cones.

### Cone Opsin Spectral Sensitivities

In the S-opsin gene, we detected the following residues at six spectral tuning sites: Phe46, Phe49, Thr52, Tyr86, Ile93, and Ala114 (GenBank accession no. KC865781). Of these, we call attention to sites 86 and 93, which have profound effects on the λ_max_ of mammalian S-opsins (Cowing et al. 2002; Parry et al. 2004; Carvalho et al. 2012). Here, the combination of Tyr86 and Ile93, which is also present in cow, indicates an S-opsin pigment with a λ_max_ of 435 nm (Cowing et al. 2002), 438 nm (Fasick et al. 2002), or 451 nm (Jacobs et al. 1998) depending on the methods used. Sequencing of exon 5 of the L-opsin gene revealed Tyr277 and Thr285, indicating an L-opsin pigment with a λ_max_ of ~562 nm (GenBank accession no. KC865780).

**Fig. 5:**
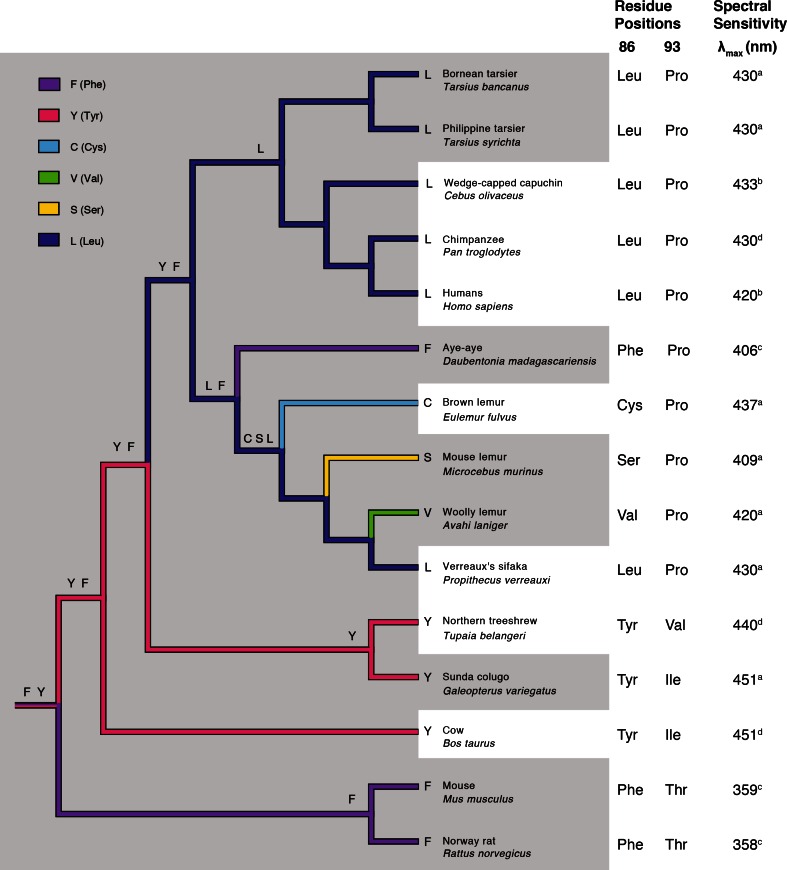
Composite tree topology of euarchontan S-opsin genes with mammalian outgroups. The λ_max_ of each S-opsin is shown on the basis of DNA sequence (^a^), microspectrophotometry (^b^), in vitro expression (^c^) or electroretinogram (^d^) (Dartnall et al. 1983; Jacobs and Neitz 1986; Neitz et al. 1991; Jacobs et al. 1996b, 1998; Yokoyama et al. 1998; Tan and Li 1999; Parry et al. 2004; Tan et al. 2005; Carvalho et al. 2012; present study). The tree shows the most probable amino acid state at each ancestral node based on their inferred likelihood at site 86 in the context of observed or inferred activity patterns among living and ancestral species (after Martin 1990). The distinction between nocturnal (*gray zone*) and diurnal (*white zone*) activity patterns conveys the prevailing hypothesis of nocturnal primate origins based principally on orbital, dental, and gross functional morphologies (Martin 1990; Cartmill 1992; Heesy and Ross 2001; Ravosa and Savakova 2004; Martin and Ross 2005; Ravosa and Dagosto 2007; Ross and Kirk 2007). The set of states at each node is ordered from most likely to least likely, excluding states with probabilities below 5 %

The ancestral primate (and euprimate) is most parsimoniously reconstructed as having an intact S-opsin gene with Phe86 or Tyr86 (Fig. 5) and Val93, Ile93, or Pro93. Estimating the ratio of nonsynonymous to synonymous rates (d_N_/d_S_ or ω) in a single parameter model (M0) on the tree shown in Supplemental Figure S3 revealed a low overall ratio of 0.39 and evidence of purifying selection across a large proportion of sites under selection and neutral models (~60 % of sites, *ω*_0_ = 0.018; Supplemental Table S1). Such values (<1) are consistent with purifying selection (a result typical of studies of genes with highly conserved functional roles).

## Discussion

The general features of the colugo eye and retina are consistent with the animal’s nocturnal activities. For instance, the relatively high rod to cone ratio and thick ONL found in colugos is typical of other nocturnal mammals, including nocturnal primates (Wikler and Rakic 1990). Similarly, nocturnal mammals have distinctive rod cell nuclei, and the inverted nuclear architecture of colugo rod cells speaks to a long history of nocturnality (Solovei et al. 2009; Perry and Pickrell 2010). Yet colugos appear to lack a tapetum lucidum (the structure responsible for the phenomenon of ‘eye shine’), as only a dark retinal pigment epithelium was evident. The limited quality of the available material precluded microscopic assessment of a potential retinal tapetum, where the retinal pigment epithelial cells contain reflective inclusions or particles. In contrast to choroidal tapeta, a retinal tapetum is rare among mammals, so far only described for fruit bats and the opossum (Ollivier et al. 2004). Unless the colugo has a retinal tapetum, the reddish to yellowish eye-shine seen in some flash photographs of colugos (Lim 2007) might be a reflection from the highly vascularized choroid, resembling the phenomenon of human ‘red eye’ in flash photos. Indeed, many truly nocturnal mammals (e.g., nocturnal rodents, Microchiroptera) lack a tapetum, so it is not an indispensable feature of nocturnal eyes. A caveat is that tapetal riboflavin may dissolve quickly in fixative solutions (for a discussion, see Kirk 2006). On the other hand, our experience with the choroidal tapeta of many nonprimate mammals is that they remain visible after long fixation.

The observation that the colugo retina is avascular also came as a surprise. The retinae of non-mammalian vertebrates are generally avascular, as are those of the monotremes and many marsupials (Chase 1982). In contrast, most placental mammals have a vascularized retina, suggesting that avascular retinae are a “primitive” feature among mammals (Chase 1982). Notably, primates and treeshrews have vascularized retinae (Johnson 1901; Müller and Peichl 1989), hence the avascularity of the colugo retina might have developed secondarily after the separation of these lineages (Janečka et al. 2007). The potential adaptive advantages of an avascular retina are unknown, but there is an obvious disadvantage: the retinal oxygen supply depends completely on choroidal circulation. Hence the thickness of the avascular retinae is limited by the diffusion distance of oxygen (Chase 1982), or the inner retina uses anaerobic metabolism (e.g., Yu and Cringle 2001; Bentmann et al. 2005). Avascular mammalian retinae have thicknesses <200 μm, whereas vascular retinae are in the range of 200–250 μm (Buttery et al. 1991). Reduced thickness is a particular problem for nocturnal species because they depend on a high rod density and hence a thick ONL for adequate low-light vision. Nocturnal mammals with vascularized retinae have 200,000–850,000 rods/mm^2^ and ONL with up to 14 tiers of rod nuclei (recent summaries: Peichl 2005; Solovei et al. 2009).

Generalizing from these findings and the observation that colugos are ‘living fossils’ (Ducrocq et al. 1992; Marivaux et al. 2006), its stands to reason that 45 million years of nocturnality might have resulted in a disabled S-opsin gene (Tan et al. 2005). Instead, we show that Sunda colugos have intact S- and L-opsin genes and that both pigments are expressed in the retina. Furthermore, we found signatures consistent with purifying selection on exon 1 of the colugo S-opsin gene. Taken together, these findings challenge the view that S-opsin genes rapidly acquire disabling mutations under nocturnal conditions, as well as the corollary view that nocturnal primates with intact S-opsin genes, such as *Daubentonia*, evolved from a diurnal ancestor (Tan et al. 2005).

Our findings also fill a crucial phylogenetic void within Euarchonta. As hypothesized by Carvalho et al. (2012), the ancestral primate (and euprimate) is most parsimoniously reconstructed as having an intact S-opsin gene with Phe86 or Tyr86 (Fig. 5) and Val93, Ile93, or Pro93. Either reconstruction indicates ten amino acid substitutions in subsequent lineages of primates and a selective aversion to UV sensitivities (360–400 nm) due to the strict conservation of Pro93 (Fig. 5). A critical test of this reconstruction would involve opsin sequence data from the pen-tailed treeshrew, *Ptilocercus lowii*, the most basal euarchontan and only nocturnal scandentian (Roberts et al. 2011). On the basis of available data, the S-opsin gene of *P. lowii*, if functional, is predicted to have Phe or Tyr at site 86 (Carvalho et al. 2012). Such findings, along with the present results, would suggest that functional S-opsin genes cannot be used as evidence of recent diurnal ancestry (*pace* Tan et al. 2005).

Yet an unanswered question remains: for nocturnal colugos, what are the potential adaptive advantages of maintaining functional S-cones? Although cones are a small minority (1–5 %) of photoreceptors in the colugo retina, they appear to be important. All mammals studied to date possess a duplex retina with both rods and cones (Ahnelt and Kolb 2000; Peichl 2005). An explanation for this pattern is that the rod pathway in the mammalian retina ‘piggybacks’ on the cone pathway and needs the cone system to convey signals to the retinal ganglion cells (review: Wässle 2004). Cone-mediated color vision is also useful when a nocturnal animal extends its activity into the dawn or dusk period, or when it is disturbed during diurnal rest. Colugos are primarily nocturnal, but they often initiate activities under dim twilight (Lim 2007), which is enriched in shorter, purplish wavelengths (Melin et al. 2012). For colugos, the presence of ‘pure’ S-cones could be advantageous for discriminating color or enhancing contrast under twilight conditions (Melin et al. 2012); however, the unusual coexpression of S- and L-opsin in many of the S-cones might impede these abilities. Another possibility, given the importance of twilight to the photoentrainment of many nocturnal species (Roenneberg and Foster 1997), is that cones with opsin coexpression contribute to irradiance detection. These issues are discussed further in Supplemental Material. We conclude by suggesting that colugos represent a promising avenue of future research for understanding the ecology and evolution of color vision among nocturnal mammals.

## Electronic supplementary material

Below is the link to the electronic supplementary material.

Supplementary material 1 (PDF 2696 kb)
