# Supplementary material for: Expression and Evolution of Short Wavelength Sensitive Opsins in Colugos: A Nocturnal Lineage That Informs Debate on Primate Origins
Source: Evol Biol. 2013 Apr 17;40(4):542–53. doi: 10.1007/s11692-013-9230-y (PMC3832777; doi:10.1007/s11692-013-9230-y)
Supplement: Supplementary file 1 — Supplementary material 1 (PDF 2696 kb) [file 11692_2013_9230_MOESM1_ESM.pdf]

Supplemental Material

Methods supplement

Opsin immunolabeling

Opsin immunolabeling followed protocols described in Peichl et al. (2005) and Schleich et al. (2010). Briefly, the tissue was preincubated for 1 h in PBS with 0.5% Triton X-100 and 10% normal goat serum (NGS), or normal donkey serum (NDS) when secondary antibodies from donkey were used. Incubation in the primary antibody/antiserum solution was for 3-4 days (free-floating tissue) or overnight (sections on the slide) at room temperature. The binding sites of the primary antibodies were detected by indirect immunofluorescence, with a 1-hour incubation in Alexa goat anti-rabbit IgG, Alexa donkey anti-goat IgG, or Alexa goat anti-mouse IgG, respectively. Double-labeling of S- and L-opsins was performed by incubating the tissue in a mixture of antisera JH 492 and sc-14363. In this case, visualization was by incubation with a mixture of Alexa 488-conjugated donkey anti-goat IgG and Cy5-conjugated donkey anti-rabbit IgG. Some retinal pieces were used for the quantification of cone densities, in which case incubation with antiserum JH 492 or JH 455 was followed by an overnight incubation in goat anti-rabbit IgG, an overnight incubation in rabbit peroxidase-anti-peroxidase (PAP) complex, and visualization with DAB and H_2O_2. Retinal pieces were flattened onto slides with the photoreceptor side up. All tissue was cover-slipped with AquaPoly/Mount.

Antibody specificity and controls

Our different immunolabeling protocols yielded consistent results. The specificity and characterization of the opsin antibodies have been described as follows. For the rod opsin antibody rho4D2, rat rod outer segments (OS) were used as immunogen, and its epitope was mapped to the rhodopsin N-terminus (Hicks and Molday 1986). The S-opsin antiserum JH 455 and the L-opsin antiserum JH 492 were raised against epitopes of the human blue and red cone opsin, respectively (Wang et al. 1992). The S-opsin marker sc-14363 is an affinity-purified goat polyclonal antibody raised against a 20-amino-acid synthetic peptide mapping within amino acids 1-50 of the human S-opsin (peptide sequence determined by Schiviz et al., 2008). Each of these antibodies has been used in a wide range of mammals and laboratories and has reliably labeled the respective photoreceptor types.

In the colugo, the rod opsin antibody rho4D2 labeled the entire photoreceptor including the soma. This pattern has been observed in the rods of many mammals, indicating that it is rod-specific in the colugo. All cone opsin labeling was localized to the photoreceptors. Controls double-labeled with both antisera specific to S-opsins, JH 455 (raised against a C-terminal epitope) and sc-14363 (raised against an N-terminal epitope), showed colocalization in the same cones. A small difference was that the sc-14363 label was restricted to the outer segment whereas JH 455 label extended to the inner segment and soma. Similarly, JH 492 labeled the outer and inner segment as well as the soma of the L-cones (see Fig. S1). We have observed this labeling pattern in a number of species and attribute it to reduced cellular preservation in tissue that had been fixed with some post mortem delay. Omission of the primary antibodies from the incubation solution resulted in no staining, showing the specificity of the secondary antibodies.

Results

General features of the eye and retina

Eye dimensions were measured in three eyes of three individuals (male / female / male; Table 1) The colugo eye resembles a slightly flattened sphere. The large thickness and diameter of the lenses indicates adaptation to dim light vision. Individual differences in eye size might be related to the unknown ages of the animals. Each retina was radially pleated and mostly detached from the fundus. We attribute this pattern to a fixation artifact, perhaps due to vitreous shrinkage in the fixative solution.

Cone density variation

Cone densities were assessed at a number of retinal positions (see Figs. S1 and S2), including a large temporal sector of one retina that extended from the optic nerve head to the periphery. This sector was PAP-DAB-labeled with a mixture of antisera JH 492 and JH 455 to guarantee strong labeling of all cones. As these antisera also labeled the cone somata (see Fig. S1), cones could be counted even in regions where some of the outer segments may have been lost during preparation. In the temporal sector, total cone densities were between 2,500 mm^{-2} and 5,400 mm^{-2}. The density variations were mostly local and appeared random. There was neither a clear peak that would indicate a central area, nor a consistent central-peripheral density gradient. In a piece from the dorsal periphery, cone densities were 7,500 – 8,000 mm^{-2}, and in a piece from the ventral periphery, 7,800 – 11,700 mm^{-2}. Hence it appears that colugo cone densities are higher in peripheral than in central retina, albeit the available tissue did not allow a complete topographical map of cone densities.

Sequence analysis

Estimating the ratio of nonsynonymous to synonymous rates (d_N/d_S or ω) in a single parameter model (M0) on the tree shown in Figure S3 revealed a low overall ratio of 0.39. Such values (<1) are consistent with purifying selection. Models allowing ω to vary across all sites were implemented to test for sites under positive selection. While the M1a/M2a comparison was significant, the frequency of sites under positive selection (ω_2 = 6.22) was only 3.5%. The Bayes Empirical Bayes (BEB) analysis in model M2a identified site 76 as the only site under positive selection. Branch models were also implemented in order to test for changes in selective constraint along the colugo lineage but no significant differences were found. In the absence of synonymous substitutions (d_S=0), PAML is unable to properly estimate ω; as a result, some of our branches lack ω value (Fig. S3; ∞, d_N is nonzero and d_S is zero).

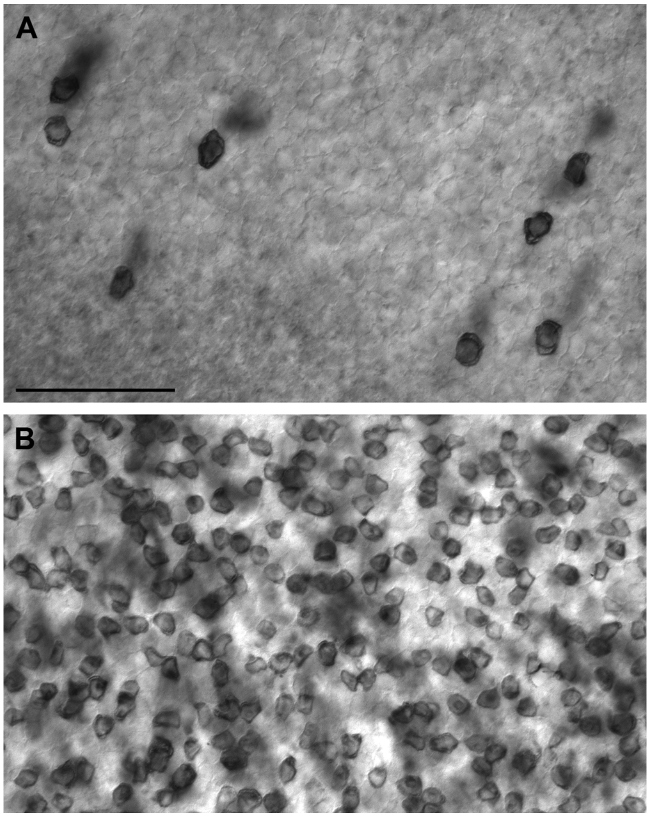

Fig. S1 Single-labeling for S-opsin **(a)** and L-opsin **(b)** by a PAP-DAB reaction in two flatmounted, closely neighboring pieces of ventral peripheral retina. The focus is on the cone somata, the out-of-focus shadows particularly evident in (a) are the labeled outer segments of the same cones. This type of material was used to assess cone densities. Scale bar 50 µm.

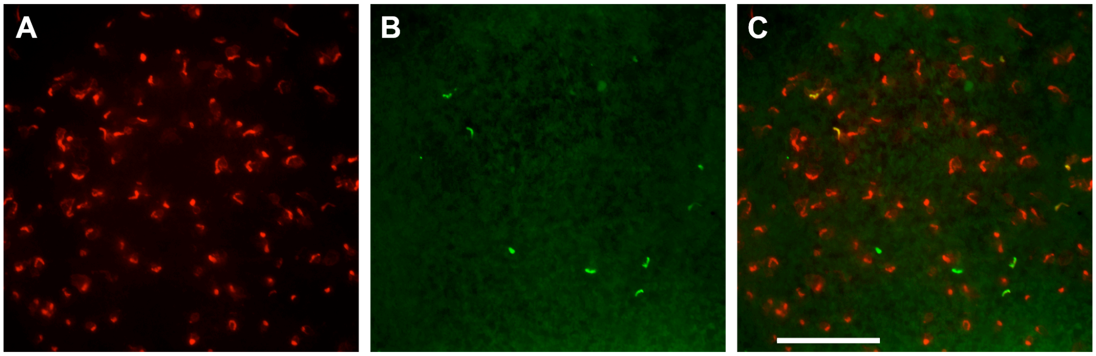

Fig. S2 Double immunofluorescence labeling for S- and L-opsin in a flatmounted piece of retina located near the optic nerve head. **a** L-opsin labeling, focus on the outer segments. **b** S-opsin labeling shows a very sparse population of cones. **c** Merge of the images in **(a)** & **(b)**. In this field, there are S-opsin-expressing cones that coexpress some L-opsin (e.g., the two cones top left that appear yellow in **(c)** and 'pure' S-cones without L-opsin (two cones at bottom middle). The level of L-opsin coexpression varies individually, it is high in the cones on top left and low in the cones on bottom right. Scale bar 50 µm.

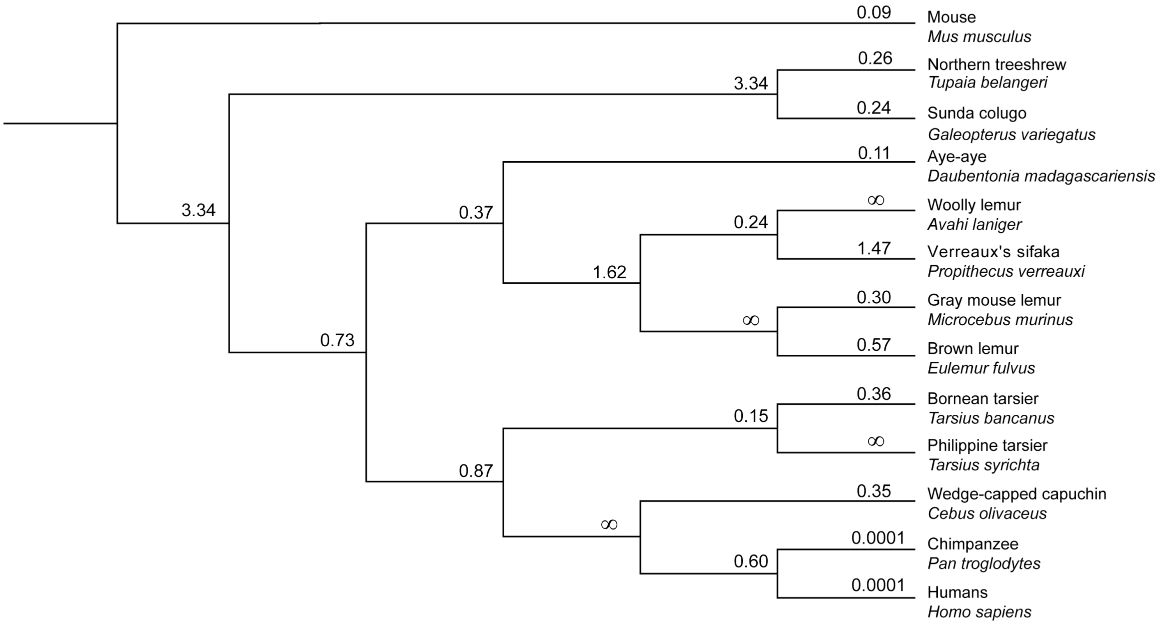

Fig. S3 Lineage-specific ω (d_N/d_S) values of exon 1 of the S-opsin across Euarchonta with a mammalian outgroup. Ratios significantly greater than 1 suggest positive selection while ratios significantly less than 1 are consistent with purifying selection on nonsynonymous mutations. Ratios non-significantly different from 1 are consistent with neutrality. ∞, d_N is nonzero and d_S is zero.

Table S1. Likelihood estimates of comparisons between evolutionary models.

**Evolutionary Model**	**Log likelihood**	**Parameter estimate**	**Likelihood Ratio Test P-value**
**Site models**			
M0	-1253.07	ω = 0.38510	N/A
M1a	-1213.97	ω_0 = 0.60376, ω_0 = 0.01927	N/A
M2a	-1208.11	ω_0 = 0.59281, ω_0 = 0.01809,<br>ω_1 = 0.37254, ω_1 = 1.00000<br>ω_2 = 0.03465, ω_2 = 6.22230*	P=0.003 (vs. M1a)
**Branch models**			
Two-ratio†	-1252.70	ω_0 = 0.40689, ω_1 = 0.24129	P=1.00 (vs. M0)

†*Galeopterus variegatus* branch selected for two-ratio model
*Positively selected amino acid site (based on *Avahi laniger* protein sequence): 76

Discussion

Colugo rods

Our estimates yield colugo rod densities of 200,000 – 250,000 mm^{-2}, and the ONL has only 6-8 tiers of photoreceptor somata. Apparently, the absence of a retinal vasculature is associated with markedly lower rod densities than found in nocturnal primates. Maximal rod densities are 325,000 mm^{-2} in the owl monkey, 450,000 mm^{-2} in the

bushbaby, and 850,000 mm^{-2} in the mouse lemur (Wikler and Rakic 1990; Dkhissi-Benyahya et al. 2001); the ONL of tarsier has 12-16 tiers of somata (Rohen 1966). On the other hand, rod densities in the avascular retina of the nocturnal to crepuscular rabbit range from a central peak of 260,000 – 308,000 mm^{-2} to a peripheral minimum of about 150,000 mm^{-2} (Young and Vaney 1991). These are closer to the colugo rod densities and suggest that, within the limitations set by avascularity, the colugo retina is also adapted to nocturnal vision. This is further evidenced by the fact that colugo rods have the inverted nuclear architecture that is a hallmark of nocturnal and crepuscular mammals (Solovei et al. 2009). Finally, the low proportion (1-5%) of cones among the photoreceptors argues for adaptation to nocturnal vision.

Colugo cone opsin co-expression and color vision

Our finding of both the S- and L-cone opsin shows that the colugo has the potential for dichromatic color vision. Dichromatic color vision based on S- and L-cones is the basic mammalian pattern, which is also maintained in most nocturnal mammals (Jacobs 1993; Peichl, 2005). Typically, S-cones form a minority (5-15%) of the cones (Szél et al., 1996; Peichl, 2005), and colugos generally conform to this pattern. In the colugo, most cones express the L-opsin, and a regionally varying minority (2–9%) of the cones expresses the S-opsin. Some of the latter are ‘pure’ S-cones, but many of them coexpress varying amounts of L-opsin. Among mammals, opsin coexpression is relatively rare, and known only from a few species of Rodentia, Lagomorpha, Insectivora and Microchiroptera (Juliusson et al. 1994; Röhlich et al. 1994; Famiglietti and Sharpe 1995; Applebury et al. 2000; Glösmann and Ahnelt 1998, 2002; Lukáts et al. 2002, 2005; Parry and Bowmaker 2002; Glösmann et al. 2008; Müller et al. 2009). The Sunda colugo can now be added to this list.

The functional significance of opsin coexpression is unclear. Coexpressed visual pigments with different spectral sensitivities can broaden the spectral sensitivity of the photoreceptor (Arikawa et al. 2003), but at the same time impede color vision. For example, the house mouse coexpresses UV- and M-opsin in a large proportion of its cones, but it can still make chromatic discriminations (Jacobs et al. 2004). Jacobs and colleagues (2004) concluded that complete segregation of the opsins into separate individual cones is not a prerequisite for color vision, but they concede that the underlying retinal color processing circuits are still obscure (Jacobs and Williams 2007). In the colugo, many of the S-cones show only relatively faint L-opsin labeling, indicating that they contain markedly less L- than S-opsin (see Figs. 4 and S2). It can be assumed that their spectral tuning is dominated by the S-opsin, making them more shortwave-sensitive than the L-cones. Hence these S-cones could still contribute significantly to color-opponent signal processing. It remains unknown whether the opsin-coexpressing cones of colugos are genuine S-cones connected to S-cone bipolar cells, but which also express some L-opsin; or whether they are generic L-cones, similar to the dual pigment cones of the mouse.

For some invertebrates, opsin coexpression can contribute to photosensory tasks such as photoentrainment (the entrainment of an organism's circadian rhythm by light to follow the daily solar cycle). For example, in the horseshoe crab, *Limulus*, the differential regulation of coexpressed opsins across light and dark cycles appears to control circadian rhythms (Katti et al. 2010). For mammals, non-visual opsins appear to be the principal means of photoentrainment (Peirson et al. 2009); however, the potential role of cone opsin coexpression has received little attention.

Literature Cited

Applebury, M. L., Antoch, M. P., Baxter, L. C., Chun, L. L. Y., Falk, J. D., Farhangfar, F., et al. (2000). The murine cone photoreceptor: A single cone type expresses both S and M opsins with retinal spatial patterning. *Neuron, 27*, 513-523.

Arikawa, K., Mizuno, S., Kinoshita, M., & Stavenga, D. G. (2003). Coexpression of two visual pigments in a photoreceptor causes an abnormally broad spectral sensitivity in the eye of the butterfly *Papilio xuthus*. *Journal of Neuroscience, 23*, 4527-4532.

Dkhissi-Benyahya, O., Szel, A., Degrip, W. J., & Cooper, H. M. (2001). Short and mid-wavelength cone distribution in a nocturnal strepsirrhine primate (*Microcebus murinus*). *Journal of Comparative Neurology, 438*, 490-504.

Famiglietti, E. V., & Sharpe, S. J. (1995). Regional topography of rod and immunocytochemically characterized "blue" and "green" cone photoreceptors in rabbit retina. *Visual Neuroscience, 12*, 1151-1175.

Glösmann, M., & Ahnelt, P. K. (1998). Coexpression of M- and S-opsin extends over the entire inferior mouse retina. *European Journal of Neuroscience, 10*, 357.

Glösmann, M., & Ahnelt, P. K. (2002). A mouse-like retinal cone phenotype in the Syrian hamster: S opsin coexpressed with M opsin in a common cone photoreceptor. *Brain Research, 929*, 139-146.

Glösmann, M., Steiner, M., Peichl, L., & Ahnelt, P. K. (2008). Cone photoreceptors and potential UV vision in a subterranean insectivore, the European mole. *Journal of Vision, 8*, 1-12.

Hicks, D., & Molday, R. S. (1986). Differential immunogold-dextran labeling of bovine and frog rod and cone cells using monoclonal antibodies against bovine rhodopsin. *Experimental Eye Research, 42*, 55-71.

Jacobs, G. H. (1993). The distribution and nature of colour vision among the mammals. *Biological Reviews, 68*, 413-471.

Jacobs, G. H., Williams, G. A., & Fenwick, J. A. (2004). Influence of cone pigment coexpression on spectral sensitivity and color vision in the mouse. *Vision Research, 44*, 1615-1622.

Jacobs, G., & Williams, G. (2007). Contributions of the mouse UV photopigment to the ERG and to vision. *Documenta Ophthalmologica, 115*, 137-144-144.

Janečka, J. E., Miller, W., Pringle, T. H., Wiens, F., Zitzmann, A., Helgen, K. M., et al. (2007). Molecular and genomic data identify the closest living relative of primates. *Science, 318*, 792-794.

Juliusson, B., Bergström, A., Röhlich, P., Ehinger, B., van Veen, T., & Szél, A. (1994). Complementary cone fields of the rabbit retina. *Investigative Ophthalmology & Visual Science, 35*, 811-818.

Katti, C., Kempler, K., Porter, M. L., Legg, A., Gonzalez, R., Garcia-Rivera, E., et al. (2010). Opsin co-expression in Limulus photoreceptors: differential regulation by light and a circadian clock. *Journal of Experimental Biology, 213*, 2589-2601.

Lim, N. T.-L. (2007). *Colugo: The flying lemur of South-East Asia*. Singapore: Draco.

Lukáts, Á., Dkhissi-Benyahya, O., Szepessy, Z., Röhlich, P., Vígh, B., Bennett, N. C., et al. (2002). Visual pigment coexpression in all cones of two rodents, the Siberian hamster, and the pouched mouse. *Investigative Ophthalmology & Visual Science, 43*, 2468-2473.

Lukáts, A., Szabo, A., Rohlich, P., Vigh, B., & Szel, A. (2005). Photopigment coexpression in mammals: comparative and developmental aspects. *Histology and Histopathology, 20*, 551-574.

Müller B., Glösmann, M, Peichl, L., Knop, G. C., Hagemann, C., & Ammermüller, J. (2009). Bat eyes have ultraviolet-sensitive cone photoreceptors. *PLoS ONE, 4*, e6390.

Parry, J. W. L., & Bowmaker, J. K. (2002). Visual pigment coexpression in guinea pig cones: A microspectrophotometric study. *Investigative Ophthalmology & Visual Science, 43*, 1662-1665.

Peichl, L. (2005). Diversity of mammalian photoreceptor properties: Adaptations to habitat and lifestyle? *The Anatomical Record Part A: Discoveries in Molecular, Cellular, and Evolutionary Biology, 287A*, 1001-1012.

Peichl, L., Chavez, A. E., Ocampo, A., Mena, W., Bozinovic, F., & Palacios, A. G. (2005). Eye and vision in the subterranean rodent cururo (*Spalacopus cyanus*, Octodontidae). *Journal of Comparative Neurology, 486*, 197-208.

Peirson, S. N., Halford S., & Foster, R. G. (2009). The evolution of irradiance detection: Melanopsin and the non-visual opsins. *Philosophical Transactions of the Royal Society B, 364,* 2849-2865.

Rohen J.W. (1966). Zur Histologie des Tarsiusauges. Albrecht Von Graefes Arch. Klin. *Experimental Ophthalmology, 169,* 299-317.

Röhlich, P., van Veen, T., & Szél, Á. (1994). Two different visual pigments in one retinal cone cell. *Neuron, 13*, 1159-1166.

Schiviz, A. N., Ruf, T., Kuebber-Heiss, A., Schubert, C., & Ahnelt, P. K. (2008). Retinal cone topography of artiodactyl mammals: Influence of body height and habitat. *Journal of Comparative Neurology, 507*, 1336-1350.

Schleich, C. E., Vielma, A., Glösmann, M., Palacios, A. G., & Peichl, L. (2010). Retinal photoreceptors of two subterranean tuco-tuco species (Rodentia, *Ctenomys*): Morphology, topography, and spectral sensitivity. *Journal of Comparative Neurology, 518*, 4001-4015.

Solovei, I., Kreysing, M., Lanctôt, C., Kösem, S., Peichl, L., Cremer, T., et al. (2009).

Nuclear architecture of rod photoreceptor cells adapts to vision in mammalian evolution. *Cell, 137*, 356-368.

Szél, Á., Röhlich, P., Caffé, A. R., & van Veen, T. (1996). Distribution of cone photoreceptors in the mammalian retina. *Microscopy Research and Technique, 35*, 445-462.

Walls G.L. (1942). *The vertebrate eye and its adaptive radiation*. Bloomfield Hills, Michigan: The Cranbrook Institute of Science.

Wang, Y., Macke, J. P., Merbs, S. L., Zack, D. J., Klaunberg, B., Bennett, J., et al. (1992). A locus control region adjacent to the human red and green visual pigment genes. *Neuron, 9*, 429-440.

Wikler, K., & Rakic, P. (1990). Distribution of photoreceptor subtypes in the retina of diurnal and nocturnal primates. *Journal of Neuroscience, 10*, 3390-3401.

Young, H.M. & Vaney, D.I. (1991). Rod-signal interneurons in the rabbit retina: 1. Rod bipolar cells. *Journal of Comparative Neurology, 310,* 139-153.
